# Lack of interaction between ErbB2 and insulin receptor substrate signaling in breast cancer

**DOI:** 10.1186/s12964-016-0148-8

**Published:** 2016-10-21

**Authors:** Susan M. Farabaugh, Bonita T. Chan, Xiaojiang Cui, Robert K. Dearth, Adrian V. Lee

**Affiliations:** 1 Women’s Cancer Research Center, Department of Pharmacology and Chemical Biology, University of Pittsburgh Cancer Institute, Magee Women’s Research Institute, 204 Craft Avenue, Room A412, Pittsburgh, PA 15213 USA; 2 Lester and Sue Smith Breast Center, Department of Molecular and Cellular Biology, Baylor College of Medicine, Houston, TX 77030 USA

**Keywords:** IRS, ErbB2, Breast cancer

## Abstract

**Background:**

ErbB2 Receptor Tyrosine Kinase 2 (ErbB2, HER2/Neu) is amplified in breast cancer and associated with poor prognosis. Growing evidence suggests interplay between ErbB2 and insulin-like growth factor (IGF) signaling. For example, ErbB2 inhibitors can block IGF-induced signaling while, conversely, IGF1R inhibitors can inhibit ErbB2 action. ErbB receptors can bind and phosphorylate insulin receptor substrates (IRS) and this may be critical for ErbB-mediated anti-estrogen resistance in breast cancer. Herein, we examined crosstalk between ErbB2 and IRSs using cancer cell lines and transgenic mouse models.

**Methods:**

MMTV-ErbB2 and MMTV-IRS2 transgenic mice were crossed to create hemizygous MMTV-ErbB2/MMTV-IRS2 bigenic mice. Signaling crosstalk between ErbB2 and IRSs was examined in vitro by knockdown or overexpression followed by western blot analysis for downstream signaling intermediates and growth assays.

**Results:**

A cross between MMTV-ErbB2 and MMTV-IRS2 mice demonstrated no enhancement of ErbB2 mediated mammary tumorigenesis or metastasis by elevated IRS2. Substantiating this, overexpression or knockdown of IRS1 or IRS2 in MMTV-ErbB2 mammary cancer cell lines had little effect upon ErbB2 signaling. Similar results were obtained in human mammary epithelial cells (MCF10A) and breast cancer cell lines.

**Conclusion:**

Despite previous evidence suggesting that ErbB receptors can bind and activate IRSs, our findings indicate that ErbB2 does not cooperate with the IRS pathway in these models to promote mammary tumorigenesis.

## Background

Insulin receptor substrate (IRS) proteins are cytoplasmic adapters which function as signaling intermediates downstream of cell surface receptors. Although IRS proteins are common intermediates of multiple growth and hormone receptors [1–6], they are most well known as signaling intermediates for the insulin receptor (InsR) and the insulin-like growth factor I receptor (IGF1R) [2, 7–9]. IRS1 and IRS2 are expressed in normal and cancerous breast epithelium [6, 8, 10]. IRS1 and IRS2 contain high homology and activate common signaling pathways, such as PI3K/Akt and MAPK/ERK kinases [11], yet these two proteins have distinct functions [12]. Generally, IRS1 is associated with tumor initiating programs such as growth and survival while IRS2 is associated more closely with progression and metastasis [13–15]. As IRS2 is the more definitive mediator of tumor progression and metastasis, we focused our studies on IRS2.

It is becoming increasingly evident that the IRS proteins are regulated by epidermal growth factor receptor (EGFR) and Erb-B2 Receptor Tyrosine Kinase 2 (ErbB2, HER2/Neu) [15–18]. For example, IRS2 levels are increased by EGFR and ErbB2 [15, 18]. Further, stimulation of cells with EGF enhances IRS phosphorylation and downstream PI3K signaling in the absence of IGF signaling [15, 19, 20]. In tamoxifen-resistant breast cancer cells, EGFR and ErbB3 recruit and phosphorylate IRS1 [16, 17].

Given the emerging evidence for interaction between EGFR/ErbB2 and IGF/IRS signaling in breast cancer, we investigated crosstalk between ErbB2 and IRSs. Herein, we demonstrate that overexpression of IRS2 in MMTV-ErbB2 transgenic mice did not alter mammary tumorigenesis or metastasis. Consistent with this, overexpression or knockdown of IRS1 and IRS2 had little or no affect upon ErbB2 action in both mouse and human mammary epithelial and breast cancer cells. Taken together, our data indicates little to no role for IRSs in ErbB2 action in breast cancer.

## Methods

### Materials

All chemicals were purchased from Sigma unless otherwise indicated. All tissue culture materials were purchased from BD Falcon and Invitrogen unless otherwise stated.

### Cell culture

The BRI-JM04 mouse cell line was maintained in DMEM (with glutamine, glucose, sodium pyruvate) with 10 % serum. The MCF-10A human mammary epithelial cell was maintained in DMEM/F-12 supplemented with 5 % horse serum, EGF, chlorea toxin, hydrocortisone and insulin according to ATCC standards.

### Transient transfection

BRI-JMO4 cells were plated at 25–30 % density one day before transfection in antibiotic free complete medium. 3.6x10^5^ cells were plated per well of a 6 well plate. For overexpression experiments, the next day, cells were transfected with pcDNA3.1 plasmids containing either HA-IRS1, HA-IRS2, or an empty vector control with Lipofectamine 2000 (Thermo Fisher Scientific). For knockdown experiments, the next day, cells were transfected with 50uM of siRNA against IRS1, IRS2, or both IRS1 and IRS2 together using DharmaFECT 1 (Dharmacon). Transfections were performed following the instructions provided by the manufacturer.

### Growth assay

Twenty-four hours after siRNA transfection, BRI-JO4 cells were washed and left to rest for four hours. 2,000 cells per well were then seeded into 96-well plates and starved overnight. After starvation, cells were treated with or without 10 % fetal bovine serum. Plates were washed with PBS and frozen on days 1 and 4. When all of the plates were collected, cell growth was examined by CyQuant (Thermo Fisher Scientific).

### Immunoblotting

Cells were lysed in RIPA lysis buffer 48 h after transfection. Protein concentration was determined using the BCA kit (Pierce). Twenty-five to 50 ug of protein was loaded onto SDS-PAGE gels for analysis. Primary antibodies of HA (Cell Signaling #3724S), pIGF-IR (Biosource #44804), IGF1R (Cell Siganling #9750), pY877-ERBB2 (Cell Signaling #2241), ERBB2 (Cell Signaling #2248), IRS1 (Upstate #06-248), IRS2 (Upstate #06-506), pAKT (Cell Signaling 9272), AKT (Cell Signaling 4060), pERK1/2 (Cell Signaling 4377), ERK1/2 (Cell Signaling 9102S) were diluted in 5 % BSA blocking buffer. Generation of ErbB2/IRS2 Bigenic Mice.

All procedures were conducted in accordance with the NIH Guide for the Care and Use of Laboratory Animals and were approved by the IACUC at Baylor College of Medicine. MMTV-ErbB2 mice were received from Jackson Laboratories [21]. MMTV-IRS2 mice have been previously reported [5]. MMTV-ErbB2 mice were maintained homozygous and MMTV-IRS were hemizygous. Mice were maintained on a 12-h light, 12-h dark schedule with ad libitum access to laboratory chow and water. To generate bigenic mice, homozygous MMTV-ErbB2 male mice were bred with hemizygous MMTV-IRS2 female mice to generate hemizygous MMTV-ErbB2 and hemizygous MMTV-ErbB2/MMTV-IRS2 bigenic mice. To study the effect of parity, hemizygous MMTV-ErbB2 and hemizygous MMTV-ErbB2/MMTV-IRS2 bigenic female mice were bred with outbred CD1 male mice and allowed to go through a full pregnancy, lactation, and involution. At weaning pups were removed and euthanized.

### Analysis of median time to tumor formation (MTTF)

Median times to tumor formation (MTTF) was determined by weekly palpation. Tumor formation was recorded when tumors were first palpable. When tumors reached 1,000 mm^3^, tumors were harvested for molecular analysis. Tumor volumes were measured with calipers and volumes were calculated using the formula Volume = (Length × Width × Width)/2.

### Histology

Five um serial tumor sections were deparaffinized, gradually hydrated, and stained for hematoxylin and eosin (H&E). Sections were then examined and scored by a pathologist to determine histological phenotypes as well as stromal, inflammatory, and lactation properties.

For the detection of lung metastases, 5 um sections were cut at intervals of 100 um through one half of the lung, all sections were stained by H&E, and then examined microscopically. Lungs were scored positive for lung metastases if they contained lesions of more than 100 cells. All lesions were stained for HA (HA-IRS2) by IHC.

## Results

### IRS2 overexpression does not affect ErbB2-mediated tumorigenesis and metastasis in transgenic mice

**Fig. 1:**
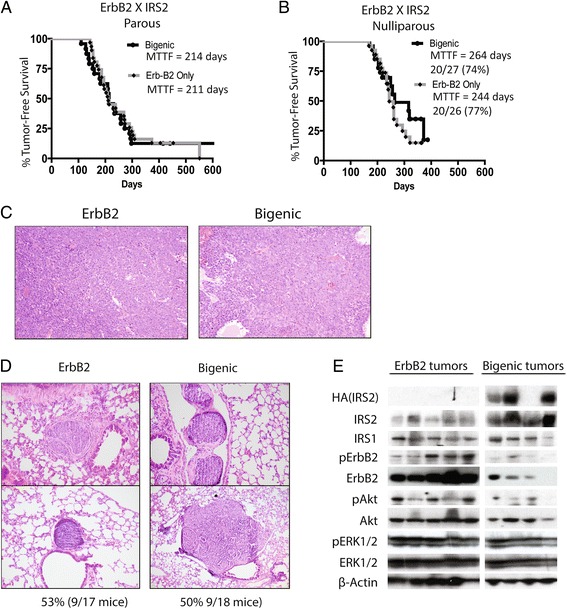
ErbB2 and ErbB2/IRS2 mice had similar time to tumor formation, tumor histology, and lung metastases. Kaplan-Meier plots of age-matched MMTV-ErbB2/IRS2 bigenic (black line) and MMTV-ErbB2 transgenic (gray line) (**a**) parous and (**b**) nulliparous mice. Mean time to tumor formation (MTTF) is shown in days and was measured by weekly palpation. Formation was recorded when first palpable. **c** H&E of tumors from nulliparous mice representing the histological phenotypes presented. **d** Immunoblotting of protein lysates from ErbB2 and ErbB2/IRS2 bigenic tumors for expression of IRS1, IRS2, HER2, and downstream signaling pathways. **e** Lungs of tumor-bearing mice were sectioned, stained with H&E, and analyzed for lung metastases. Numbers represent percentage of mice containing lung lesions to total number of mice analyzed

Recent evidence suggests that ErbB2 may utilize IRSs for signaling. To determine potential cooperation between these pathways, we analyzed the effect of elevated IRS2 on ErbB2-mediated breast tumorigenesis. We compared mammary tumorigenesis between MMTV-ErbB2 and MMTV-ErbB2/MMTV-IRS2 bigenic mice. Under parous (p) and nulliparous (np) conditions, median time to tumor formation (MTTF) was similar for both ErbB2 (*p* = 214 and np = 244 days) and ErbB2/IRS2 bigenic mice (*p* = 211 and np = 264 days) (Fig. 1a and b). Histological analysis revealed that the bigenic ErbB2/IRS2 tumors closely mirrored the phenotype of ErbB2 tumors with 88 % solid adenocarcinoma in ErbB2 and 66 % solid adenocarcinoma in ErbB2/IRS2 bigenic tumors (Fig. 1c and Table 1). Although some bigenic tumors (21 %) did show features of MMTV-IRS2 mouse tumors such as squamous differentiation [5], most bigenic tumors recapitulated ErbB2 tumor phenotypes, suggesting that the ErbB2 pathway may be the primary driver of tumorigenesis in these bigenic mice. We also noted no difference in lung metastasis in ErbB2 and bigenic ErbB2/IRS2 mice with nodules observed at 53 and 50 % for ErbB2 and bigenic mice, respectively (Fig. 1d and Table 1). Protein analysis of the tumors confirmed overexpression of HA-tagged IRS2 in the bigenic ErbB2/IRS2 tumors (Fig. 1e). No difference in IRS1 levels were noted. Interestingly, ErbB2 levels were decreased in bigenic tumors.

**Table 1:** Histological analysis of the ErbB2/IRS2 bigenic tumors closely mirror ErbB2 single transgenic tumors

	ErbB2	ErbB2/IRS2
	25 Tumors-18 mice	24 Tumors-17 mice
Histological Types		
Solid Adenocarcinoma	88 % (22/25)	66 % (16/24)
Differentiated		
Squamous Carcinoma	0	4 % (1/24)
Adenosquamous Carcinoma	8 % (2/25)	21 % (5/24)
Pillary	0	4 % (1/24)

Histological analysis of nulliparous and parous tumors were combined for a total of 24 ErbB2/IRS2 bigenic tumors (18 mice) and 25 age-matched ErbB2 transgenic tumors (17 mice). All tumor phenotypes were scored by a pathologist based on H&E staining

### Modulation of IRS1 and IRS2 levels in ErbB2-expressing mouse cells has little effect upon ErbB2 signaling and cell growth

**Fig. 2:**
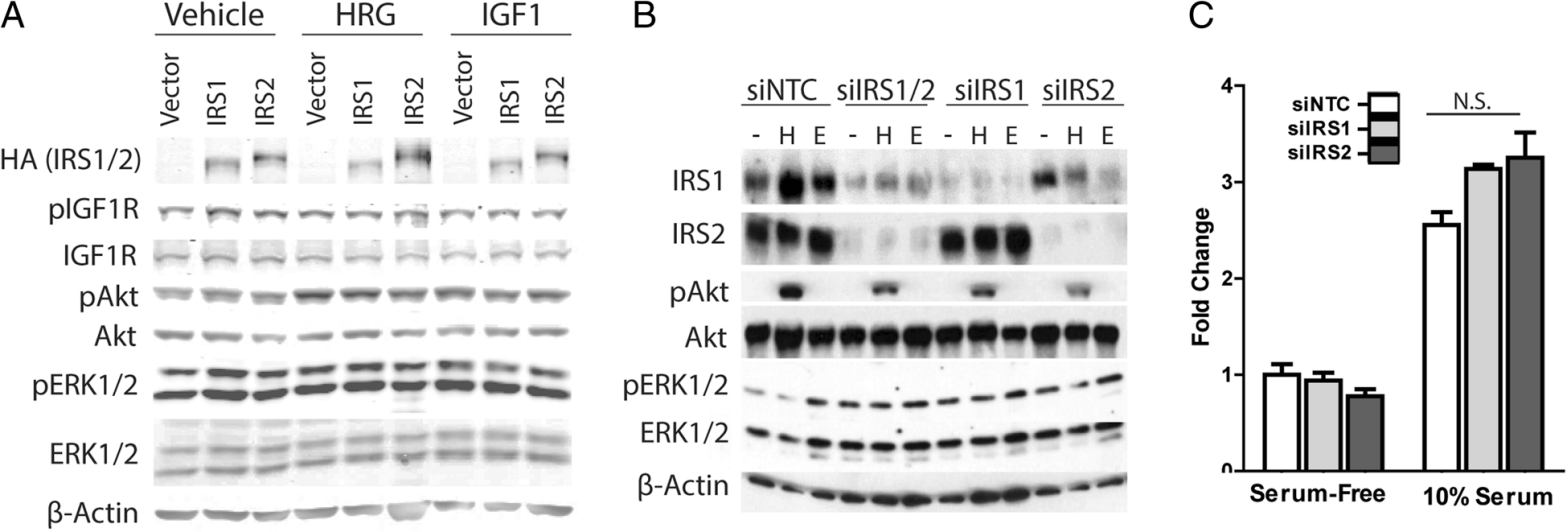
Modulation of IRS1 and IRS2 levels in ErbB2 expressing mouse cells alters ErbB2 signaling but not cell growth. **a** BRI-JM04 mouse cells were starved overnight and then treated in the morning with either 60 ng/ul of heregulin (HRG) or 20 ng/ul of IGF1 for 15 mins and then harvested. Expression of ErbB2 signaling proteins was determined by immunoblotting. Blots are representative of 3 experiments. **b** BRI-JM04 cells were transfected with siRNA against IRS1, IRS2, or both IRS1 and IRS2. At 48 h post-transfection, cells were stimulated with 20 ng/ul heregulin (H) or 60 ng/ul EGF (E) for 15 min. Cells were then harvested and analyzed for ErbB2 signaling by immunoblotting. **c** After IRS1 or IRS2 knockdown, BRI-JMO4 cells were plated and maintained with or without serum for 4 days. Cell growth was determined by CyQuant. Graph is representative of 3 experiments. One-way Anova was applied. N.S. = not significant

As overexpression of IRS2 showed an unexpected lack of influence on ErbB2-driven tumorigenic phenotypes (Fig. 1), we further evaluated the interaction using in vitro culture models. As the functions and roles for IRS1 and IRS2 are still unclear, we analyzed the effect of both IRSs. To determine modulation of ErbB2 signaling upon overexpression of IRS1 and IRS2 in vitro, HA-tagged IRS1 or HA-tagged IRS2 were transiently transfected into the MMTV-ErbB2 mouse cell line BRI-JM04. Knowlden et al. demonstrated that, in ER+ cells, heregulin (HRG) stimulates the recruitment of IRS1 to ErbB3 [16, 17] and thus we examined the effect of HRG in BRI0-JM04 cells with or without IRS overexpression. As shown in Fig. 2a, overexpression of IRS1 or IRS2 in BRI-JM04 cells in SFM (vehicle) conditions did not affect phosphorylation of IGF1R, Akt, or ERK1/2. HRG and IGF1 treatment increased phosphorylation of Akt and ERK1/2 in BR10-JM04 cells, but overexpression of IRS1 or IRS2 (noted by HA expression) had little to no effect upon HRG or IGF1 induced Akt or ERK1/2 phosphorylation. If anything, we noted a reproducible but very small decrease in HRG-induced Akt activity.

To determine if IRS1 or IRS2 are required for ErbB2 signaling, we knocked-down each IRS individually and together in ErbB2 expressing BRI-JM04 cells and then stimulated cells with HRG or EGF. siRNA knockdown of IRS1 and IRS2 were both efficient and specific. In control cells, HRG induced p-Akt, whereas EGF induced p-ERK1/2. Knockdown of IRS1, IRS2 or the combination caused a slight decrease in HRG-induced p-Akt. No major differences were observed with EGF treatments with or without IRS1/IRS2 knockdown. We next tested whether the minor effect of IRS knockdown on p-Akt may reduce cell growth; however, knockdown of IRS1 or IRS2 followed by a growth assay showed no significant effect on cells grown in SFM or 10 % serum (Fig. 2c).

### Elevated IRS1 and IRS2 levels in human breast cells does not affect EGFR or ErbB2 signaling

**Fig. 3:**
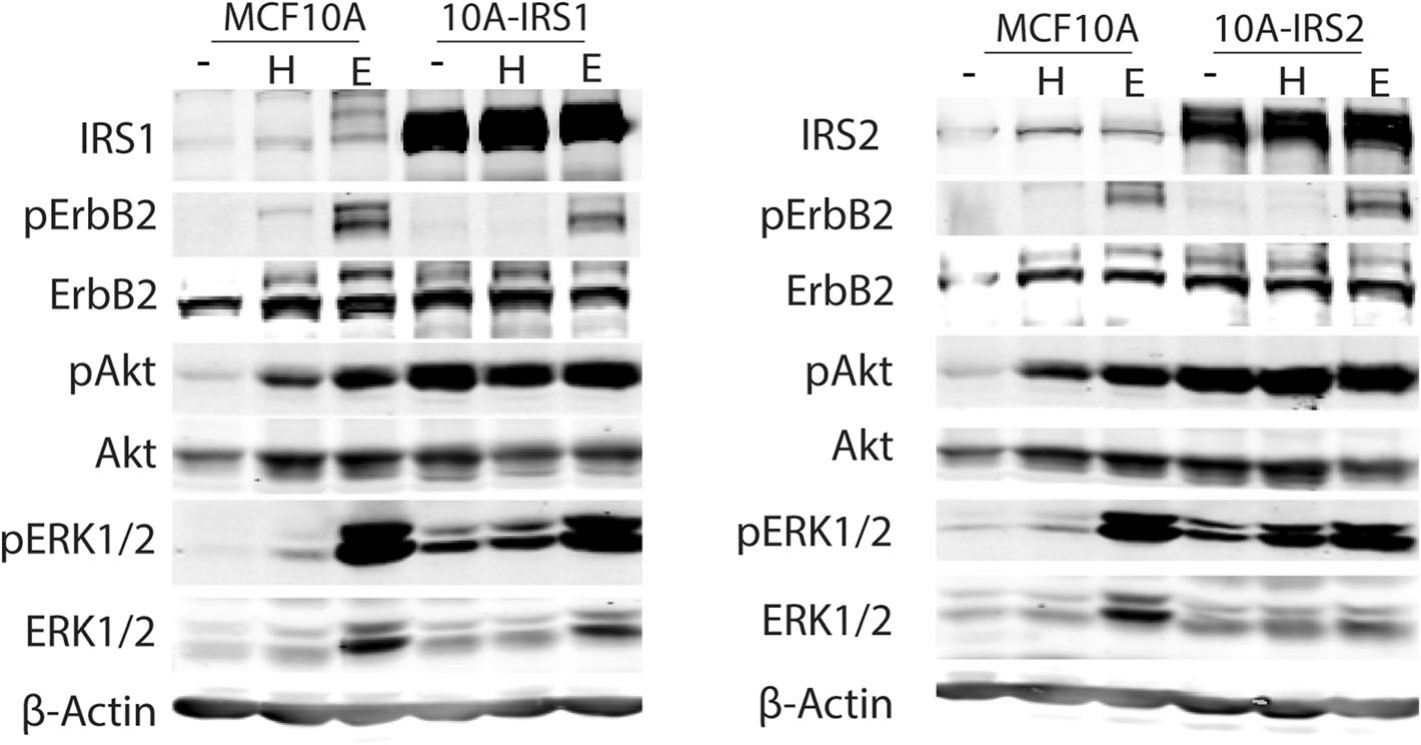
Modulation of IRS1 and IRS2 expression in human MCF10A breast cells alters ErbB2 signaling. MCF10A cells stably overexpressing HA-IRS1 or HA-IRS2 were serum starved for 24 h and then treated with 20 ng/ul heregulin (H) or 60 ng/ul EGF (E) for 15 mins and harvested. Immunoblotting was performed to determine ErbB2 signaling

As the previous experiments were all performed using murine models, we explored the effect of IRS1 and IRS2 on ErbB2 signaling in human immortalized non-transformed human mammary epithelial cells (MCF10A) which overexpress IRS1 or IRS2 and have previously been reported to have enhanced IGF signaling [5]. Stimulation of MCF10A cells with HRG or EGF resulted in phosphorylation of ErbB2, Akt and ERK1/2. Cells overexpressing IRS1 or IRS2 had increased basal phosphorylation of Akt that was not affected by HRG or EGF treatment. The basal phosphorylation of ERK1/2 was also elevated by IRS overexpression, but in this case the induction (both fold induction and total phosphorylated levels) by EGF and HRG was lower than in MCF10A cells (Fig. 3).

## Discussion

The IGF and ErbB pathways are involved in tumor initiation, tumor progression and resistance to therapy [22–25]. These pathways overlap in their signal transduction pathways, sharing PI3K/Akt and MEK/ERK signaling, and several studies suggest crosstalk between the pathways may be at least partially responsible for resistance to both ErbB2- and IGF1R-targetered therapies [25–31]. Several studies have previously demonstrated that EGFR/ErbB recruits and activates IRSs [16–18], and in some cases suggests a process for therapeutic resistance to drugs such as tamoxifen. In this report we examined the role of IRSs in ErbB2 action in vitro and in vivo. Contrary to expected, we demonstrate that increased IRS1 or IRS2 expression does not enhance the ability of ErbB2 to further stimulate downstream signaling pathways and enhance tumorigenic phenotypes.

In this study, we hypothesized that increasing both ErbB2 and IRSs would result in enhanced downstream signaling (through Akt and ERK1/2). However, in our in vitro cell line data, we observed that increased or decreased IRS levels had little to no effect upon ErbB2 signaling. It should be noted that our studies were performed in the presence of overexpressed ErbB2 which was developed to mimic the amplification seen in human breast cancer. Other studies have noted ErbB2-IRS interaction and cooperation in antiestrogen resistance where the ErbB2 is simply elevated and not amplified. It is possible that in our model systems, ErbB2 is overexpressed at a level where it no longer requires IRS expression beyond what is endogenously expressed or the level at which ErbB2 is able to induce. This does not rule out a role for the ErbB2-IRS cooperation in antiestrogen resistance, but suggests that ErbB2 amplified human breast cancers may have sufficient ErbB2-induced regulation and signaling. It is also possible that the overexpression of ErbB2 has disrupted normal signaling networks and interactions. Recent studies have shown an intricate network of feedback mechanisms with PI3K/S6K regulating IGF1R/IRSs and MEK negatively feeding back to the ErbB receptors [32, 33]. There is thus a fine balance of receptor signaling components with interference possible when one is overexpressed. Limited amounts of substrates can also cause squelching. For example, recruitment of IRSs to alternate receptors such as the EGFR family limits the amount of IRS available for association with IGF1R which in turn limits downstream IGF1R signaling [16]. Inhibition of the EGFR pathway can then direct signaling back through the IGF1R-IRS association [16].

In vivo, overexpression of IRS2 did not alter ErbB2 mediated tumorigenesis. Interestingly, ErbB2 tumors showed similar histologic phenotypes (solid adenocarcinomas) compared to bigenic ErbB2/IRS2 tumors, suggesting that even in the presence of elevated IRS2, the ErbB2 signaling pathway remains the major driver of tumorigenesis. We did identify a small amount of squamous metaplasia in bigenic tumors, something that is rarely seen in ErbB2 tumors, but widely observed in IRS2 tumors; however, this effect was minimal and rarely seen.

## Conclusion

In conclusion, we find little evidence that increased IRS expression enhances ErbB2 mediated signaling and tumorigenesis. This does not corroborate or conflict with other studies as our models may have sufficient IRS expression to support ErbB2 signaling. We show this phenotype in both human and murine models, in vitro and in vivo. A better understanding of this complex systems network will be critical to optimize response to anti-growth factor receptor and signaling intermediate (e.g. PI3K, mTOR) inhibitors in breast cancer.

## Abbreviations

EGF (E): Epidermal growth factor
EGFR: Epidermal growth factor receptor
ErbB2: Erb-B2 receptor tyrosine kinase 2
H&E: Hematoxylin and eosin
HRG (H): Heregulin
IGF1R: Insulin-like growth factor receptor 1
IHC: Immunohistochemistry
InsR: Insulin receptor
IRS: Insulin receptor substrate
MMTV: Mouse mammary tumor virus
MTTF: Median time to tumor formation
